# Sex specific cognitive differences in Parkinson disease

**DOI:** 10.1038/s41531-020-0109-1

**Published:** 2020-04-08

**Authors:** Tyler Harrison Reekes, Christopher Ian Higginson, Christina Raye Ledbetter, Niroshan Sathivadivel, Richard Matthew Zweig, Elizabeth Ann Disbrow

**Affiliations:** 1 Department of Pharmacology, Toxicology, and Neuroscience, Louisiana State University Health Sciences Center, Shreveport, LA USA; 2 LSU Health Shreveport Center for Brain Health, Shreveport, LA USA; 3 Department of Psychology, Loyola University of Maryland, Baltimore, MD USA; 4 Department of Neurosurgery, Louisiana State University Health Sciences Center, Shreveport, LA USA; 5 Department of Neurology, Louisiana State University Health Sciences Center, Shreveport, LA USA

**Keywords:** Neurological manifestations, Parkinson's disease

## Abstract

Parkinson disease (PD) is a progressive neurodegenerative disorder that is 1.5 times more common in males than in females. While motor progression tends to be more aggressive in males, little is known about sex difference in cognitive progression. We tested the hypothesis that there are sex differences in cognitive dysfunction in non-demented PD. We evaluated 84 participants (38 females) with PD and 59 controls (27 females) for demographic variables and cognitive function, including attention, working memory, executive function, and processing speed. Multivariate ANOVA revealed no significant differences between groups for demographic variables, including age, years of education, global cogntition, daytime sleepiness, predicted premorbid IQ, UPDRS score, PD phenotype, or disease duration. For cognitive variables, we found poorer performance in males versus females with PD for measures of executive function and processing speed, but no difference between male and female controls. Specifically, PD males showed greater deficits in Verbal Fluency (category fluency, category switching, and category switching accuracy), Color Word Interference (inhibition), and speed of processing (SDMT). There were no differences in measures of working memory or attention across sex and inconsistent findings for switching. Our data indicate that males with PD have significantly greater executive and processing speed impairments compared to females despite no differences in demographic variables or other measures of disease severity. Our findings are consistent with the steeper slope of disease progression reported in males with PD.

## Introduction

Parkinson disease (PD) is a progressive neurodegenerative disorder traditionally characterized by motor signs^1^; however, cognitive dysfunction has been shown in patients even in the absence of Parkinson disease dementia (PDD), including impairments in executive function^2–4^, processing speed^5–7^, and spatial working memory^8,9^. In addition, PD is associated with increased risk for progressive cognitive decline from mild cognitive impairment (MCI) to dementia^8^. Prevalence estimates vary, but MCI affects nearly a fourth of PD patients, and dementia eventually affects over 80% of patients with 20-year survival^10^.

PD is 1.5 times more common in males than in females^11^. There is evidence that symptomatic PD onset is delayed in females^12,13^, with females reporting fewer symptoms in the pre-clinical phase of PD. Females often develop a more benign PD tremor dominant (TD) phenotype^12^ (67% compared to 48% in males) associated with less severe motor deterioration and localized basal ganglia degeneration as opposed to more widespread disease. In contrast, studies have shown that males more often present with a postural instability dominant phenotype including gait disturbances (PIGD), freezing of gait (56% males), and falling (59% males)^14,15^. Interestingly, the TD phenotype has been associated with less cognitive dysfunction^16,17^, while PIGD is associated with greater deficits in executive function^18–20^.

Sex differences across several cognitive domains have been described in healthy aging. Healthy aging males have been shown to outperform females in tasks of visuospatial functioning; however, females performed significantly better in most other tasks of cognition^21–23^. Furthermore, males had significantly steeper rates of decline across several cognitive domains, while females showed no greater decline across any measure^21^. Sex differences in cognitive performance have been reported for brain-related diseases as well. For example, Weiss et al.^24^ found that, in patients with a range of psychiatric disorders, males outperformed females on tests assessing visuospatial ability while females performed better on tasks involving verbal acuity. This difference in affected cognitive domain was maintained across disease type^23,25^.

Cognitive decline in PD is associated with advanced age, disease progression, and male sex^11,26^; however, data on differences in cognitive impairment between males and females with PD is sparse. For example, Jankovic and Kapadia^27^ found that males showed a steeper slope of decline on all subscales of the UPDRS, including UPDRS I, which measures mentation, behavior, and mood. This scale contains a single “mentation” question which requires the investigator to rate intellectual impairment on a scale of 0–4, with 1 = mild (consistent forgetfulness with partial recollection of events and no other difficulties) and 4 = severe (severe memory loss with orientation preserved to person only. Unable to make judgements or solve problems. Requires much help with personal care. Cannot be left alone at all). Males declined more rapidly than females on the entire UPDRS I scale, though the slope of decline was less pronounced on UPDRS I than on UPDRS II (activities of daily living) or III (motor performance). However, other studies have found no differences in motor progression^28,29^ and in early, untreated PD patients, females have shown poorer cognitive performance than males^30^.

Thus, there is a paucity of data describing sex differences in cognitive function, especially across specific cognitive domains. Therefore, we tested the hypothesis that there are sex differences in cognitive dysfunction in non-demented PD. We examined the domains of attention, working memory, verbal fluency, inhibition, switching, and processing speed. Identifying sex differences in cognitive dysfunction may improve prediction, diagnosis, and early treatment of cognitive dysfunction in PD and has the potential to shed light on mechanisms of neuroprotection.

## Results

### Group differences

Multivariate ANOVA (MANOVA) revealed that there were no significant differences across sex in either control or PD groups for demographic variables including age (*F*(3, 139) = 0.928, *p* = 0.429), years of education (*F*(3, 139) = 0.647, *p* = 0.586), global cognition (Mini-Mental State Examination (MMSE)) score (*F*(3, 139) = 0.396, *p* = 0.756), daytime sleepiness (Epworth Sleepiness Scale, ESS) (*F*(3, 139) = 2.179, *p* = 0.093), or NART-R (*F*(3, 139) = 1.573, *p* = 0.199). For the Geriatric Depression Scale (GDS) there were significant differences between control and PD groups; however, within disease category, there were no significant differences between sexes (*F*(4, 138) = 0.950, *p* = 0.331; Table 1). In the control groups there were no significant sex differences on any cognitive outcomes.

**Table 1:** Demographic variables.

	*N*	Age (years)	Education (years)	MMSE	ESS	NART-R	GDS^*^
Control (M)	32	65.63 (5.84)	16.53 (3.29)	28.94 (1.01)	7.25 (3.59)	112.03 (9.28)	2.75 (3.07)
Control (F)	27	65.04 (6.93)	16.59 (3.13)	28.59 (1.39)	6.33 (3.37)	113.83 (5.44)	2.82 (3.89)
PD (M)	46	67.34 (6.45)	15.83 (2.68)	28.83 (1.25)	8.46 (4.14)	113.18 (7.77)	6.15 (5.58)
PD (F)	38	66.53 (5.97)	16.50 (2.46)	28.84 (1.31)	8.63 (5.07)	115.53 (6.36)	4.76 (3.94)

^*^Significant differences between control and PD groups, *p* < 0.05; no significant differences between sexes in control and PD groups.

In assessing motor performance across the PD groups, there were no differences by sex in dosage of dopamine equivalents (*F*(1, 76) = 0.260, *p* = 0.612). Furthermore, there were no significant differences between sexes on any UPDRS subscale scores (Table 2), total score (*F*(1, 74) = 0.311, *p* = 0.579), or H&Y scale score (*F*(1, 73) = 0.469, *p* = 0.496), nor were there differences in disease duration (*F*(1, 68) = 1.744, *p* = 0.191). Finally, the proportion of TD versus PIGD phonotypes did not significantly differ between the sexes, *X*^2^ (2, *N* = 75) = 0.010, *p* = 0.995. Therefore, there were no significant differences in demographic variables, nor were there any significant differences between clinical presentation or disease severity across the sexes.

**Table 2:** PD disease severity, dopamine equivalents and UPDRS scores in PD groups.

	Dopamine equivalent (mg)	H&Y (median)	UPDRS I	UPDRS II	UPDRS III	UPDRS IV	UPDRS Total
PD (M)	591.05 (484.88)	1.88 (0.84)	2.44 (1.91)	8.97 (5.38)	20.08 (12.80)	4.09 (2.95)	36.22 (18.26)
PD (F)	648.68 (499.56)	2.02 (0.80)	1.88 (1.64)	9.34 (7.06)	22.16 (13.43)	5.44 (4.08)	38.81 (22.29)

In tests of attention and working memory there were no significant differences across sexes in the PD group for digit span forward (*F*(1, 83) = 0.106, *p* = 0.745) or backward (*F*(1, 83) = 1.202, *p* = 0.276) (Table 3). Executive functions were measured via the D-KEFS Verbal Fluency (VF), Color Word Interference (CWI), and Trail Making (TMT) tests. In the PD group, we found sex differences in VF category fluency (*F*(1, 82) = 11.820, *p* < 0.001; Cohen’s *d* = 0.76), category switching (*F*(1, 82) = 10.855, *p* < 0.001; Cohen’s *d* = 0.72), and category switching accuracy (*F*(1, 82) = 6.026, *p* = 0.016; Cohen’s *d* = 0.54; Table 3). These data show that males with PD produced fewer words per category and fewer switches between categories compared to females. For CWI we found differences between sexes in the PD group on the inhibition measure (*F*(1, 82) = 4.286, *p* = 0.042; Cohen’s *d* = 0.46) but not on the inhibition switching condition (*F*(1, 82) = 0.801, *p* = 0.374; Table 4). Variability was high for inhibition switch. PD males took longer to complete the inhibition test than females. On the Trail Making Test, using condition 5 as a motor speed correction, we found a trend toward deficits in males switching ability (condition 4 minus 5; *F*(1, 82) = 3.151, *p* = 0.080; Table 5). Finally, for speed of processing, there were significant differences between sexes in PD on the SDMT (*F*(1, 86) = 4.824, *p* = 0.031; Cohen’s *d* = 0.48; Table 5). Males with PD completed fewer items on the SDMT in 90 s compared to females.

**Table 3:** WAIS-III digit span forward (DSF) and backward (DSB) and D-KEFS Verbal Fluency (VF) scores for category fluency, category switching, and total switching accuracy.

	DSF	DSB	Letter fluency	Category fluency*	Category switch*	Total switch accuracy*
Control (M)	10.19 (2.47)	7.41 (2.24)	37.5 (11.04)	39.5 (7.56)	13.19 (3.29)	11.47 (3.81)
Control (F)	9.96 (2.08)	6.56 (2.26)	37.22 (9.48)	42.26 (7.36)	13.93 (2.97)	11.96 (3.56)
PD (M)	9.96 (2.32)	6.50 (1.63)	40.52 (13.08)	35.72 (9.33)	11.87 (2.79)	10.37 (3.30)
PD (F)	10.13 (1.71)	7.00 (2.14)	43.50 (12.18)	42.06 (7.14)	13.91 (2.86)	12.21 (3.57)

*Significant differences between sexes in PD group, *p* < 0.05.

**Table 4:** D-KEFS Color Word Interference (CWI) scores.

	Color naming	Word naming	Inhibition*	Inhibition switch
Control (M)	32.14 (6.25)	22.86 (4.42)	61.11 (16.00)	65.57 (15.07)
Control (F)	29.73 (5.19)	22.30 (2.85)	60.86 (11.38)	64.76 (12.46)
PD (M)	35.25 (7.95)	25.73 (6.07)	73.82 (26.03)	81.97 (29.00)
PD (F)	33.26 (7.85)	24.09 (4.99)	63.65 (17.00)	75.78 (34.44)

*Significant differences between sexes in PD group, *p* < 0.05).

**Table 5:** D-KEFS Trail Making Test (TMT) scores and SDMT scores.

	Condition 1^**^	Condition 2	Condition 3*	Condition 4	Condition 5	4 minus 5**	SDMT*
Control (M)	26.90 (13.15)	37.11 (15.02)	35.13 (16.51)	94.32 (38.99)	26.40 (8.97)	67.91 (38.34)	51.21 (7.94)
Control (F)	25.22 (6.95)	34.71 (9.09)	35.94 (11.74)	83.12 (35.62)	31.02 (11.71)	52.11 (36.69)	54.56 (8.14)
PD (M)	34.89 (12.89)	47.81 (19.18)	51.71 (21.62)	122.87 (56.33)	46.03 (49.12)	76.84 (51.43)	42.28 (10.94)
PD (F)	30.22 (8.65)	44.84 (18.73)	42.01 (15.38)	103.37 (52.55)	46.05 (26.07)	57.32 (48.59)	47.92 (12.58)

^**^Trending differences between sexes in the PD group, *p* < 0.08. *Significant differences between sexes in the PD group, *p* < 0.05.

### Normative data and effect size

When comparing normed mean scores from disease and sex-based groups, a pattern emerged. In general, females and controls showed similar performance while males showed lower, usually below average scaled scores (Table 6). However, all scores tended to be in the normal range, within 1 SD of the mean. Groups in our study did not vary by age or years of education. The mean education level for all participants was relatively high (approximately 16 years) and scaled scores from group means are consistently slightly above average for controls.

**Table 6:** Scaled scores for all reported measures.

	Control (M)	Control (F)	PD (M)	PD (F)
VF letter fluency	11	10	12	12
VF category fluency*	12	13	10	13
VF category switch*	11	12	9	12
VF total switch accuracy*	10	11	9	11
CWI color naming	10	11	9	10
CWI word naming	11	11	10	10
CWI inhibition*	10	10	7	9
CWI inhibition switch	12	12	9	10
TMT condition 1^**^	10	11	7	9
TMT condition 2	12	12	10	10
TMT condition 3*	12	12	10	11
TMT condition 4	11	12	9	11
TMT condition 5	12	11	9	9
SDMT*	Above average	Above average	Below average	Average

Scaled scores for all reported measures. For SDMT, the mean score for subjects > 55 is 47.3 items for the oral test (Sheridan et al.^61^). **Trending differences between sexes in the PD group *p* < 0.08. *Significant differences between sexes in the PD group, *p* < 0.05.

We calculated Cohen’s *d* to evaluate effect size. We identified small (0.2–0.5) and medium (0.5–0.8) effect sizes^31^. The verbal fluency measures had the largest effect sizes (0.5–0.8), while SDMT (0.48), trail making (0.39), and inhibition (0.46) were small to medium. Interpreting these differences in the context of the tests themselves, we found that the difference in average letter fluency was approximately three responses, for category fluency was approximately six responses, and for switch number was about two switches. Similarly, for SDMT the average difference in items completed across groups was approximately six items. The difference in average completion time for the inhibition task was about 10 s and trail making (condition 4–5) was about 20 s.

## Discussion

Males with PD had poorer performance on cognitive measures of verbal fluency, inhibition, and processing speed compared to females, a difference that was not observed in healthy controls and could not be accounted for by demographic or disease variables. Switching measures showed higher variability and differences across sex were not significant. Nor were there differences in measures of working memory or attention. Thus, males showed consistently poorer performance across multiple cognitive domains.

Sex differences in cognitive function in PD are consistent with previous work on cognitive decline to dementia. For example, Cereda et al.^26^ found that male gender was a risk factor for dementia in PD. Similarly, a study by Cholerton et al.^32^ showed that male sex was a predictor of cognitive decline from no impairment to MCI as well as from MCI to PDD. Furthermore, males had a more rapid progression of cognitive decline in processing speed and working memory^32^. We extend this work by showing baseline sex differences in multiple cognitive domains for people with PD with MMSE scores in the normal range. Interestingly, Gao et al.^33^ reported that cognitive disturbances were more severe in Chinese females than in males with PD. Females presented with significantly lower scores on the MOCA (males = 23.8 vs. females = 20.6) after adjustment for disease duration and years of education, and females had a significantly lower level of education (males = 11.3 vs. females = 8.2 years), which may explain this discrepancy. In our data, both global cognitive function and years of education were similar among males and females with PD.

One factor that has been proposed to explain sex differences in cognitive aging is related to cognitive reserve. Cognitive reserve, or an individual’s ability to compensate for increasing brain pathophysiology, has been shown to develop through a combination of experiences through life, such as educational and occupational attainment^34–36^. Cognitive reserve has been shown to contribute to delayed detection of AD and other related dementias^37–40^. It has been proposed that the increased incidences of dementia in females may be related to reduced cognitive reserve^41^. It is known that differences between males and females are associated with both biological factors, including chromosomal and hormonal differences, and sociocultural differences between groups such as environmental (e.g. toxicant) exposures, occupation, and educational expectations and access^42,43^.

The fact that historically females received less educational access than males has been referred to as “the educational gender gap”^44^. While the gender gap has been largely overcome in education, an elderly cohort like ours could have been subject to gender disparities in educational access and attainment^45^. However, in both control and PD groups we found no sex differences in predicted premorbid IQ or years of education. It is also universally maintained that there are no significant differences between males and females with respect to IQ^46^. Furthermore, occupation and educational achievement have been most closely associated with IQ^47^. Here we demonstrate that in PD, males show consistently greater deficits in cognitive function than females even though the groups were matched for several variables that are associated with cognitive reserve. Thus, cognitive reserve is not likely to contribute significantly to the observed sex differences in cognitive performance.

Two PD phenotypes have been described based on motor signs: PIGD dominant and TD. PIGD is more common in men^12^, and is associated with increased disease severity, including more profound balance and gait disturbance, bradykinesia and cognitive impairments compared to patients presenting with TD^12,16–20,27,48^. In contrast, TD is more common in women and is associated with earlier age of onset, less cognitive impairment, and slower overall progression of the disease^17,27,48^.

Burn et al.^17^ described a faster rate of cognitive decline in patients with PIGD motor subtype. Furthermore, Jankovic and Kapadia^27^ found that PIGD was associated with a steeper annual rate of global decline compared to TD in areas of mentation, behavior and mood, activities of daily living, and motor performance. Recently, Kelly et al.^18^ found that deficits in global cognition, specifically executive function, memory, and phonemic fluency, were associated with PIGD symptom severity. Moreover, they found that executive function deficits were associated with gait disturbance, freezing, and postural stability impairments^18^.

Our finding that men are more cognitively impaired than women appears in agreement with this phenotypic profile, assuming that PIGD is more common in men and is associated with greater cognitive decline. PD symptomatology reflects dopaminergic as well as more widespread Lewy body pathology, and males may be more vulnerable to the widespread pathology that affects gait and cognitive function associated with the PIGD phenotype^7^. In our sample, the proportion of TD/PIGD phenotype was similar in the male and female groups based on a subset of UPDRS items, indicating that phenotype likely does not account for the cognitive difference between males and females observed here. However, our sample size was likely too small (<20 per group in some cases) and underpowered to make a definitive statement on a sex × phenotype interaction.

Our data suggest that non-demented male patients with PD represent a disease subgroup that is more vulnerable to cognitive impairment. Furthermore, we found that sex is more strongly associated with cognitive performance than motor phenotype. Identification of disease subgroups is an important step in the understanding and treatment of the disease.

## Methods

### Participants

A total of 84 participants (38 females) with PD without dementia and 59 participants (27 females) without PD were recruited from movement disorder clinics, senior centers, support groups, and veteran’s organizations. Information about the study was provided to potential participants in the form of group presentations and/or fliers, and interested individuals contacted the lab to volunteer. Self-reported sex was used to divide participants into male and female groups. Only male and female categorizations were reported. Participants were between the ages of 54 and 83 years. An inclusion criterion was PD previously diagnosed by a movement disorder neurologist with a positive history of pharmacological intervention with responses to levodopa and/or dopamine agonists (PD only). Other inclusion criteria were native English speaking and between the ages of 50 and 85. Exclusion criteria were traumatic head or spine injury, history of stroke, brain tumor, drug abuse, and global cognitive impairment (Mini-Mental State Exam score < 24). Additional variables, which were considered potential confounds, included GDS, Epworth Sleepiness Scale (ESS), Premorbid IQ (NART-R), and dopamine equivalents. Participants with PD were administered the Unified Parkinson’s Disease Rating Scale (UPDRS) to assess disease stage and symptom severity at the time of evaluation. All motor and cognitive testing was performed when participants with PD were at their best ON medication state, for example, after his/her morning dose. This study was approved by an institutional review board at University of California, Davis and Louisiana State University Health Sciences Center, Shreveport. All participants provided written informed consent. Data were collected between 5 October 2010 and 1 December 2017.

### Instruments

Demographic and disease state data, motor performance, as well as a battery of neuropsychological tests surveying a range of cognitive domains were collected from each participant. Cognitive domains tested included attention and working memory, executive function, and processing speed. Each participant was tested in a private examination room by a trained psychological technician. Time of testing ranged from 2–4 h and the tests were pseudorandomized across participants.

### Descriptive measures

- *Mini-Mental State Examination* (MMSE)^49^: This test is a commonly used instrument to gauge global level of cognitive function in areas of orientation, registration, attention and calculation, recall, and language.
- *Epworth Sleepiness Scale* (ESS)^50^: This test is a self-evaluation of daytime sleepiness used to gauge the propensity for sleep during eight daytime activities. Daytime sleepiness has been closely associated with PD, and has been shown to be associated with a steeper rate of cognitive decline than patients without daytime sleepiness^51^.
- *North American Adult Reading Test, Revised* (NAART-R)^52^: This test is a measure used to predict premorbid intellectual function. As a measure of semantic memory, it is resistant to decline. The test requires examinees to pronounce irregularly spelled words.
- *Geriatric Depression Scale* (GDS)^53^: This test is a self-reported measure of depression symptoms in elderly persons. It consists of 15 yes/no questions.
- Dopamine equivalents were calculated according to Tomlinson et al.^54^.
- *The Unified Parkinson’s Disease Rating Scale* (UPDRS)^55^: This test is a clinical scale used to determine the severity of Parkinson Disease. Areas surveyed include: (1) mentation, behavior, and mood; (2) activities of daily living, and (3) motor performance. The UPDRS interview and clinical evaluation was administered by a nurse practitioner or a trained technician. Specific questions from the UPDRS were used to determine TD or PIGD phenotypes^56^.
- *Hoehn and Yahr Scale* (H&Y)^1^: This scale is a measure of PD disease progression. A score of one through five is given with higher scores representing a more advanced disease stage.

### Attention and working memory

- *WAIS-III Digit Span*^57^: This measure is divided into two separate tasks, digit span forward (DSF) and digit span backwards (DSB). DSF requires the participant to verbally reproduce a given sequence of single-digit numbers. DSB requires the participant to retain and manipulate a given sequence but reproduce the sequence in reverse order. Both tasks use progressively longer sequences until the sequence can no longer be reproduced. DSF is considered a test of simple auditory attention, and DSB is considered a test of auditory working memory.

### Executive function

- *The Delis-Kaplan Executive Function Scale (D-KEFS) Verbal Fluency (VF) Test*^58^: This test measures verbal orthographic and semantic fluency through a series of three conditions: letter fluency, category fluency, and category switching. The letter fluency condition consists of three trials where participants are asked to state as many words as possible beginning with a specific letter for 1 min each. Category fluency consists of two trials where a category is provided and the participant states as many words within that category for 1 min each. Finally, category switching is a single 1-min trial in which participants are required to switch between providing exemplars of two different categories. Total switching accuracy is the number of correct switches between categories.
- *The D-KEFS Color Word Interference Test (CWI)*^58^: This test includes four conditions: color naming, word reading, inhibition, and inhibition/switching. Color naming requires the participant to name the color of sequential blocks as quickly as possible. Word reading requires the participant to read color words printed in black text as quickly as possible. The inhibition task requires the participant to state the color of the ink in which a color word is printed, inhibiting the overlearned word reading response. Finally, the inhibition/switching task requires the participant to either state the ink color of the word, or if the word is located inside of a box, then the participant must switch and read the word instead of naming the ink color, thereby adding a cognitive flexibility element to the task.
- *The D-KEFS Trail Making Test (TMT)*^58^: This test measures cognitive flexibility through a series of five conditions: visual scanning, number sequencing, letter sequencing, number–letter switching, and motor speed. In the scanning condition participants cross out targets in an array of distractors to assess visual scanning difficulties that can impact performance on later conditions. The number and letter sequencing trials involve drawing lines to connect numbers then letters in order. These two conditions are included to ensure that sequencing abilities are intact prior to the switching condition in which participants must connect both letters and numbers in order but switching back and forth between the two categories (i.e., 1-A-2-B…) assessing cognitive flexibility. A final motor speed condition in which participants use a pencil to follow dashed lines is administered to assess general psychomotor slowing. This psychomotor slowing condition is subtracted from the switching condition (condition 4 minus 5) in order to isolate switching (cognitive flexibility).

### Processing speed

- Symbol Digit Modalities Test (SDMT, Oral version)^59^: This test is a measure of processing speed. The participant is presented with a decoded key containing corresponding symbols and numbers. The task is to correctly code as many symbols as possible in 90 s by verbally stating the number that goes with each symbol in order in a series of rows of randomly ordered symbols below the key.

### Power analysis

Preliminary data on cognitive sex differences in PD were sparse, making it difficult to perform a meaningful sample size estimate. However, we were able to confirm adequate power for the current study using the SDMT data. We found that for 80% power with an alpha level of 5%, 32 subjects per group were required, which is in line with the reported results.

### Group comparisons

Data from all subjects who met inclusion and exclusion criteria were included in the analysis. Multivariant analysis of variance (MANOVA) was used to assess differences in demographic and descriptive variables across sex within disease category (healthy controls vs. PD). One-way ANOVA was used to assess differences between the sexes in PD subjects. All tests were two-tailed. To determine disease phenotype, calculations were performed based on Stebbins et al. using UPDRS scores^56^, and chi-squared analysis was used to determine significant sex differences in the frequency of the phenotypes. Lastly, effect size for significantly different comparisons was calculated as Cohen’s *d*.

To control for alpha inflation, we used MANOVA, which reduces family-wise error without assuming independence of the dependent variables (e.g. researchgate.net). Each MANOVA is considered single comparisons. In addition, justification for use of uncorrected alpha level = 0.05 comes from Ridker et al.^60^, who did not correct for multiple comparisons in a similar situation where the same basic question (in our case sex differences in cognition) was asked multiple times and all results pointed to the same conclusion.

### Reporting summary

Further information on experimental design is available in the Nature Research Reporting Summary linked to this article.

## Supplementary Information

Supplementary information

Reporting summary

## Data Availability

Data are available from the corresponding author, given reasonable requests.
